# Genomic and phenotypic characterization of non-O157 Shiga toxin-producing *Escherichia coli* from Bangladeshi cattle reveals diverse virulence and resistance profiles

**DOI:** 10.1128/spectrum.00148-26

**Published:** 2026-06-22

**Authors:** Tahrima Saiha Huq, Fahad Khan, Aura Rahman, Fariza Shams, Noshin Ibnat Rib, Abdus Sadique, Jahidul Alam, Syeda Naushin Tabassum, Rayhan Imam, Ananya Saha, Md Aminul Islam, Arman Hossain, Saiful Islam, Kamruzzaman Rumman, Sudhakar Bhandare, Lovleen Tina Joshi, Md Tanvir Rahman, Munirul Alam, Md Bashir Uddin, Maqsud Hossain

**Affiliations:** 1 NSU Genome Research Institute (NGRI), North South University 54495, Dhaka, Bangladesh; 2 Department of Biochemistry & Microbiology, School of Health & Life Sciences, North South University 54495, Dhaka, Bangladesh; 3 Department of Cell and Molecular Biology, Tulane University 5783, New Orleans, Louisiana, USA; 4 International Bioscience and Oncology Research (IBSOR), Dhaka, Bangladesh; 5 School of Veterinary Medicine and Science, University of Nottingham 6123, Nottingham, United Kingdom; 6 School of Medicine, University of Limerick 8808, Limerick, Ireland; 7 Department of Microbiology and Hygiene, Faculty of Veterinary Science, Bangladesh Agricultural University 54492, Mymensingh, Bangladesh; 8 icddr, b (International Centre for Diarrheal Disease Research, Bangladesh) 56291 , Dhaka, Bangladesh; 9 Department of Medicine, Sylhet Agricultural University 399806, Sylhet, Bangladesh; Newcastle University, Newcastle Upon Tyne, United Kingdom

**Keywords:** non-O157 STEC, antimicrobial resistance, virulence factors, zoonotic pathogens, cattle, Shiga toxin

## Abstract

Shiga toxin-producing *Escherichia coli* (STEC) are important zoonotic pathogens that cause diarrheal diseases and hemolytic uremic syndrome in humans, but data from Bangladesh remain limited. We collected 352 rectal swabs and 2 retail meat samples from cattle at the Gabtoli Hat livestock market (Dhaka, Banglades) in 2017–2018 and recovered 1 *E. coli* isolate per sample (*n* = 354). PCR screening detected *stx1* and/or *stx2* in 228/354 isolates (64.4%): 77 carried *stx1* only, 68 carried *stx2* only, and 83 carried both genes. Antimicrobial susceptibility testing revealed frequent resistance to mecillinam, tetracyclines, macrolides, and piperacillin, while resistance to ciprofloxacin, gentamicin, and colistin was uncommon, and no isolate displayed imipenem resistance. To explore the genetic diversity, eleven non-O157 isolates (including five *stx*-positive representatives stratified by toxin profiles, phylogroups, and resistance phenotypes) were sequenced with Illumina MiSeq. All genomes lacked the canonical intimin gene *eae;* however, they universally encoded *eaeH*, type 1 fimbrial genes, and *rpoS*. Comparative phylogenomics against 212 publicly available *stx*-positive genomes placed several cattle isolates near human and food-associated lineages, suggesting shared lineage relationships rather than direct transmission. Phenotypically, all sequenced isolates formed weak-to-moderate biofilms, and only two showed marked survival in normal human serum, indicating virulence-associated heterogeneity independent of *iss* copy number. Collectively, these results show that cattle in Bangladesh carry a notable reservoir of virulence and resistance-associated traits relevant to public health. These findings underscore the need for integrated One Health–based genomic surveillance, limited antibiotic use in livestock, and targeted public health interventions to reduce human transmission risk.

**IMPORTANCE:**

Genomic surveillance of non-O157 Shiga toxin-producing *Escherichia coli* in cattle from a livestock market in Dhaka, Bangladesh, is limited. By screening cattle-derived *E. coli* isolates*,* we found that nearly two-thirds of isolates carried Shiga toxin genes and that resistance to commonly used antibiotics was common. Whole-genome sequencing of 11 selected isolates revealed diverse non-O157 lineages that lacked the classical intimin gene but carried alternative adhesion factors, stress-response genes, and diverse plasmid and prophage content. Several cattle-derived isolates showed genetic relatedness to lineages observed in human and food-associated strains, although direct transmission cannot be inferred without matched epidemiological and temporal data. These findings highlight the value of urban livestock markets within an integrated One Health approach, supporting genomic surveillance, prudent antibiotic use in livestock, and targeted public health interventions to reduce the risk of human transmission.

## INTRODUCTION

Shiga toxin-producing *Escherichia coli* (STEC) is one of the major zoonotic pathogens responsible for a range of gastrointestinal illnesses, including diarrhea and hemolytic uremic syndrome (HUS), which can lead to kidney failure and can be fatal (1, 2). Cattle and other ruminants serve as primary reservoirs of STEC, which can be transmitted to humans through contaminated food, direct animal contact, or environmental exposure (3, 4). O157:H7, among the various STEC types, has been extensively studied due to its high pathogenicity and association with outbreaks (5). However, non-O157 STEC strains are increasingly recognized as significant public health threats, with rising detection rates attributed to improved surveillance techniques (3, 6).

*E. coli* is an extremely diverse bacterial species that can be categorized into three major groups based on disease-causing capability: commensal or nonpathogenic *E. coli*, intestinal pathogenic *E. coli* (such as STEC), and extraintestinal pathogenic *E. coli* (ExPEC) (7). ExPEC is further subclassified into uropathogenic *E. coli* (UPEC), sepsis-associated *E. coli* (SEPEC), and neonatal meningitis-associated *E. coli* (MNEC) (8). Although STEC infections are widely documented globally, the prevalence and genomic characterization of non-O157 STEC in Bangladesh remain poorly defined. Addressing this knowledge gap is crucial for understanding local transmission dynamics. Previous studies have reported a high prevalence of *E. coli*-associated infections in Bangladesh, particularly in human cases of urinary tract infections (7).

Several Bangladeshi studies have demonstrated that cattle are an important reservoir of *stx*-encoding Shiga toxin genes. A landmark study by Islam et al. (9) reported a high prevalence of genetically diverse STEC among slaughtered cattle in Dhaka, including both O157 and multiple non-O157 serogroups carrying *stx1, stx2*, and other virulence factors (9). This provides strong regional evidence that STEC circulate widely in local livestock populations. However, despite these findings, the genomic characteristics and antimicrobial resistance (AMR) profiles of non-O157 STEC in Bangladeshi cattle remain unexplored. Hossain et al. (10) identified the widespread presence of multidrug-resistant *E. coli* strains in clinical samples, highlighting the potential risk of horizontal gene transfer of resistance determinants between human and animal populations (7).

Globally, six non-O157 major STEC serogroups—O26, O45, O103, O111, O121, and O145—have been found responsible for foodborne infections, demonstrating genetic and virulence similarities to O157 strains (11). Notably, non-O157 STEC infections have been linked to severe clinical outcomes, including outbreaks in Europe and North America (2, 12). In Sweden, specific non-O157 serotypes have been associated with bloody diarrhea and severe illness, underscoring the need for comprehensive genomic characterization to understand their virulence potential (2).

In Bangladesh, limited studies have investigated the prevalence and genomic characteristics of STEC. While *E. coli* has been recognized as a dominant pathogen in human infections (7), there is a knowledge gap regarding its presence and pathogenicity in livestock. Given the frequent and close contact between humans and cattle in Bangladesh, zoonotic transmission of virulent and antimicrobial-resistant STEC strains represents a significant public health concern. Previous findings on multidrug-resistant *E. coli* in clinical settings suggest that cattle-associated strains could serve as reservoirs for antimicrobial resistance genes, posing a public health risk (7).

The increasing threat of antimicrobial resistance among STEC strains further complicates treatment options. Several studies have reported multi-drug resistance (MDR) in STEC isolates, often attributed to antibiotic misuse in livestock production (13, 14). Horizontal gene transfer mechanisms, such as conjugative plasmids, facilitate the spread of resistance genes, enhancing the resilience of these pathogens against broad-spectrum antibiotics (15, 16). Recent investigations, including those from Bangladesh and other low- and middle-income countries, underscore the urgent need for regional antimicrobial surveillance and stewardship programs (9).

Despite the recognized public health importance of STEC, genomic data on non-O157 cattle-derived *E. coli* from Bangladesh remain scarce. In this study, we used PCR-based screening, antimicrobial susceptibility testing (AST), selected phenotypic assays, and whole-genome sequencing of a diversity-enriched subset of isolates to characterize stx carriage, resistance patterns, virulence-associated genes, and broader genomic relationships among non-O157 *E. coli* isolated from Bangladeshi cattle. By comparing the sequenced isolates with publicly available stx-positive genomes, we aimed to define their phylogenetic context and genomic diversity. These findings provide baseline evidence to support future genomic surveillance and One Health–oriented risk assessment in Bangladesh.

## MATERIALS AND METHODS

### Study design, setting, and ethics

This cross-sectional study was conducted between 2017 and 2018 at Gabtoli Hat (Dhaka, Bangladesh), a major livestock market. All sampling and experimental procedures complied with institutional ethical standards and were approved by the Sylhet Agricultural University Institutional Animal Care and Use Committee (IACUC; approval #AUP2017025). Experimental work was carried out collaboratively between the NSU Genome Research Institute (NGRI), North South University, Dhaka, and the Department of Medicine, Sylhet Agricultural University, Sylhet, Bangladesh.

### Sample collection and molecular detection of STEC in cattle

During 2017–2018, a total of 354 cattle-derived samples were collected across multiple sampling rounds. Rectal swabs were obtained using sterile, pre-moistened swabs inserted approximately 3–5 cm into the rectum. Following enrichment and plating, up to three morphologically distinct colonies were initially subcultured from each SMAC plate to capture observable colony heterogeneity. For downstream prevalence analysis, however, a single representative *E. coli* isolate per sample was retained to avoid duplication of isolates with identical toxin and serotype profiles originating from the same specimen. Accordingly, the reported stx-positive proportion should be interpreted as an isolate-based estimate derived from one representative colony per sample, rather than as a comprehensive measure of within-sample strain diversity. This resulted in 354 unique cattle-derived isolates (352 from feces and 2 from meat), which are deposited in Figshare (https://doi.org/10.6084/m9.figshare.32148352). Sorbitol-negative colonies (typically associated with potential O157:H7) were prioritized, but sorbitol-positive colonies were also retained to capture non-O157 STEC diversity. SMAC was therefore used both as a selective/differential medium to detect potential O157 strains and as a general recovery medium for *E. coli*. All selected colonies were sub-cultured on Luria-Bertani (LB) agar to obtain pure isolates before downstream analyses.

In addition to rectal swabs, two retail cattle meat samples were collected during the same period; these yielded isolates A35 and A36. Swabs and meat rinses were enriched overnight in Luria-Bertani (LB) broth (37°C, ~16 h, static), streaked onto SMAC plates, and processed using the same colony-selection procedures described above. These two meat samples were included as supplementary specimens and were not intended to support prevalence estimation or formal source-specific comparison. We also included blank swabs and broth-only enrichment controls in every sampling round to ensure there was no cross-contamination during enrichment or plating. None of these controls showed any stx-positive amplification.

### Phenotypic virulence assays

Antimicrobial susceptibility testing (AST) was carried out on a descriptive subset of 65 isolates using the Kirby–Bauer disk diffusion method on Mueller–Hinton agar (MHA) at 35–37°C, following the CLSI M100 guidelines (17). The 65 isolates were selected to capture diversity across sampling rounds, Shiga toxin profiles, and source types while remaining feasible within available laboratory resources. Accordingly, this subset was not randomly selected, and the AST findings are presented as descriptive results for the tested subset rather than prevalence estimates for the full 354-isolate collection. *Escherichia coli* ATCC 25922 was used as the quality-control strain (18). Susceptibility categories (susceptible, intermediate, and resistant) were interpreted according to CLSI criteria, with “intermediate” reported exactly as defined in the standards (17).

### Biofilm formation assay

Biofilm formation was quantified using a modified version of a previously described protocol (19). Briefly, 2 μL of overnight LB cultures were inoculated into 198 μL of fresh LB in sterile 96-well microtiter plates, with four technical replicates per strain. Wells containing only LB served as blanks. Plates were incubated overnight at 37°C, and optical density (OD) was recorded at 600 nm to measure bacterial growth (19). Following incubation, cultures were discarded, wells were rinsed twice with sterile water, air-dried, and stained with 250 μL of 0.1% crystal violet for 15 min. Excess stain was removed, and the bound dye was solubilized in 200 μL of glacial acetic acid (19). Biofilm biomass was quantified by measuring OD at 560 nm and normalized to growth (OD_600_) to calculate the standard biofilm formation (SBF) index (19). Strains were categorized as weak, moderate, or strong biofilm formers using the Stepanovic criteria (19).

### Serum resistance assay

Serum resistance was assessed as described previously (10) with minor modifications. Overnight cultures were mixed in a 1:9 (vol/vol) ratio with normal human serum and incubated at 37°C. Viable bacteria were quantified at 0 and 3 h by serial dilution and plating to determine colony-forming units. Percent survival was calculated as (CFU at 3 h/CFU at 0 h) × 100. Heat-inactivated serum was included as a control. The use of human serum for this study was reviewed and approved by the Institutional Review Board (IRB approval no. 2020/OR-NSU/IRB/1205).

### Serotyping and PCR screening

Latex agglutination serotyping for *E. coli* O157 was performed following the manufacturer’s protocol (Oxoid, UK). Presumptive O157-positive isolates were subjected to confirmatory PCR targeting the O157 O-antigen gene rfbE and the H7 flagellin gene fliC(H7). None of these isolates were positive for either marker, indicating that no true O157:H7 strain was present. Consistent with this, none of the 11 isolates subsequently selected for whole-genome sequencing (WGS) carried an O157 O-antigen locus, and all WGS-derived serotypes were non-O157. Together, these findings indicate that the latex reactions represented false positives in this collection.

Genomic DNA was extracted from a single colony grown overnight at 37°C in LB broth using the QIAGEN Mini Kit (QIAGEN, Germany) following the manufacturer’s protocol, and its purity was confirmed with a NanoDrop 2000 Spectrophotometer (Thermo Fisher Scientific, USA). Conventional PCR targeted *stx1*, *stx2, eae*, and *fliC* genes was performed using published primers and cycling conditions (20). PCR-based phylogrouping was performed using the Clermont et al. (21) quadruplex scheme, employing the published primer sets (chuA, yjaA, TspE4.C2, and the internal control arpA) and the recommended cycling protocol (21). PCR phylotyping was used to assign preliminary phylogroups prior to genome sequencing, while *in silico* phylogrouping (ClermonTyping web tool) was used for confirmation after assembly. Positive controls (*E. coli* O157:H7 EDL933 for *stx1*, *stx2*, and *eae*) and negative controls (MG1655 and no-template controls) were included in all PCR runs.

### Selection of isolates for WGS

PCR screening showed that 64% of the 354 cattle-derived samples carried *stx* genes. These prevalence estimates are based on the initial PCR screen of all samples and are not derived from the subset selected for WGS. From this larger collection, we selected 11 isolates for whole-genome sequencing. The selection was deliberate, with the goal of capturing as much genetic and phenotypic diversity as possible rather than estimating prevalence. For this reason, the chosen isolates represent all observed toxin profiles (*stx1*, *stx2*, and dual carriage), different phylogroups identified through PCR, and both rectal swab and meat sources. Of the 11 sequenced isolates from our study, 5 carried stx genes, and 6 were stx-negative.

### Illumina sequencing and *in silico* characterization

Library preparation was carried out using the NEBNext Ultra II DNA Library Prep Kit (New England Biolabs, USA) following the manufacturer’s protocol. Whole-genome sequencing (WGS) of the isolates was performed on the Illumina MiSeq platform at the NSU Genome Research Institute (NGRI), generating 2 × 150 bp paired-end reads. Reads were quality-filtered and assembled with SPAdes; contigs <200 bp and/or <20× coverage were removed. Genome assembly quality metrics, including the number of contigs and *N*_50_ values, were assessed using QUAST (22) and are available in Figshare (https://doi.org/10.6084/m9.figshare.32148352). Genome annotation was performed with RASTtk v2.0 (23). Serotype and plasmid content were inferred using the CGE tools (Serotype Finder v2.0 and Plasmid Finder/pMLST v2.0) with thresholds ≥90% identity and ≥60% coverage unless otherwise noted (24). AMR genes were identified with ResFinder v4.1 and CARD v3.2.9 (25). Virulence factors were detected with VirulenceFinder v2.0 and BLASTn against VFDB, using ≥90% identity and ≥60% coverage thresholds (25, 26).

Sequence types were assigned from assemblies using the Achtman seven-locus MLST (27) scheme with mlst v2.25.0 (Torsten Seemann) against PubMLST. Isolates without exact allele matches were reported as unassigned, and putative novel alleles were exported using the --novel option for follow-up.

The PubMLST allele and profile databases were downloaded and formatted locally using the mlst-make_blast_db utility. Sequence format normalization was handled using any2fasta (version 0.6.1), and BLAST searches were executed using BLAST+ (NCBI BLAST, version ≥2.12). The *iss* locus was screened by BLASTn against a curated reference sequence (GenBank accession AF042279.1). Putative *iss* homologs meeting identity/coverage thresholds were aligned with MAFFT and visualized in Jalview (28, 29).

### Phylogenomics and accessory-genome comparison

A total of 212 publicly available *E. coli* genomes carrying Shiga toxin genes (*stx*) and one reference genome (*E. coli* O157:H7 strain EDL933) were retrieved from the NCBI database; the full list is available in Figshare (https://doi.org/10.6084/m9.figshare.32148352). These, along with the 11 genomes generated in this study, were aligned using Snippy (30) to construct a core-genome SNP alignment. The EDL933 chromosome sequence (GenBank accession NC_002655.2) was used as the reference for SNP calling.

A maximum-likelihood phylogenetic tree was inferred using FastTree (31) with the GTR model, midpoint-rooted, and branch support was assessed using SH-like local support values. The tree was visualized using the Interactive Tree of Life (iTOL) platform (32). Pairwise SNP distances were calculated from the same core SNP alignment and are provided in the Figshare data set. To compare the accessory-genome structure among the isolates, Roary (33) was performed. The pairwise SNP-distance matrices, Roary outputs, accessory-genome tree, pangenome summary files, and analysis scripts have been uploaded to Figshare.

### Mobilome characterization and resistance determinant profiling

The studied genomes were analyzed to characterize the mobilome components and resistance-associated gene content, including plasmids, conjugation systems, prophage-like regions, integrons, and nonvirulence resistance determinants. Plasmid content was reconstructed from draft assemblies using MOB-suite v3.0.1 (34) (MOB-recon and MOB-typer) with reference databases. Plasmid mobility potential and predicted mating-pair formation (MPF) systems were inferred using MOB-typer (34), and plasmid incompatibility groups were identified independently using PlasmidFinder (35) via ABRicate v1.2.0.

To assess horizontal transfer potential beyond reconstructed plasmids, conjugation-associated systems were detected using CONJScan (36) models implemented in MacSyFinder (37). For each isolate, outputs were summarized as the number of detected systems and the inferred MPF types. Integrons were screened using IntegronFinder (December 2025) (38). The presence or absence of integrons, integrase genes (intI), and attC sites was recorded.

Prophage-like regions were identified using two complementary approaches. PhiSpy (39) was applied to Prokka-annotated (40) assemblies to estimate prophage number and total length. In parallel, Phigaro v2.4.0 (41) was used on contigs ≥20 kb to quantify prophage burden, which was defined as the total predicted prophage DNA for integrated mobilome analyses.

Resistance-associated features were identified using NCBI AMRFinderPlus v4.2.5 run on nucleotide assemblies with the --plus option enabled. The AMRFinderPlus reference database was updated immediately prior to analysis using amrfinder -u, and the database version used was 2025-12-03.1. AMRFinderPlus (42) outputs were parsed per isolate. For the mobilome analysis, only AMRFinderPlus hits with a defined resistance class were included. Hits annotated as virulence factors or lacking a clear resistance category were excluded, so the final resistance set included antimicrobial, biocide, and metal tolerance determinants only. Mobilome and resistance outputs were integrated and analyzed in Python using pandas. Associations among mobilome features were explored using Spearman rank correlations, and differences between stx-positive and stx-negative isolates were assessed using two-sided Mann–Whitney *U*-tests, interpreted descriptively due to limited sample size.

## RESULTS

### Prevalence of STEC in cattle-derived isolates from Dhaka, Bangladesh

From January 2017 to December 2018, we conducted 9 sampling rounds at Gabtoli Hat and collected 352 bovine fecal samples and 2 cattle meat samples, which are detailed in the publicly available data set (Figshare: https://doi.org/10.6084/m9.figshare.32148352). Latex agglutination initially flagged 57 isolates as presumptive *E. coli* O157. However, confirmatory PCR of this latex-positive subset detected neither the O157-specific rfbE gene nor fliC(H7) in any isolate, indicating that these represented presumptive latex false positives rather than confirmed O157:H7. None of these 57 were included among the 11 isolates selected for whole-genome sequencing (WGS). Confirmatory screening of the latex-positive set found no evidence of O157, indicating that the latex assay produced false positives in this collection.

**Fig 1:**
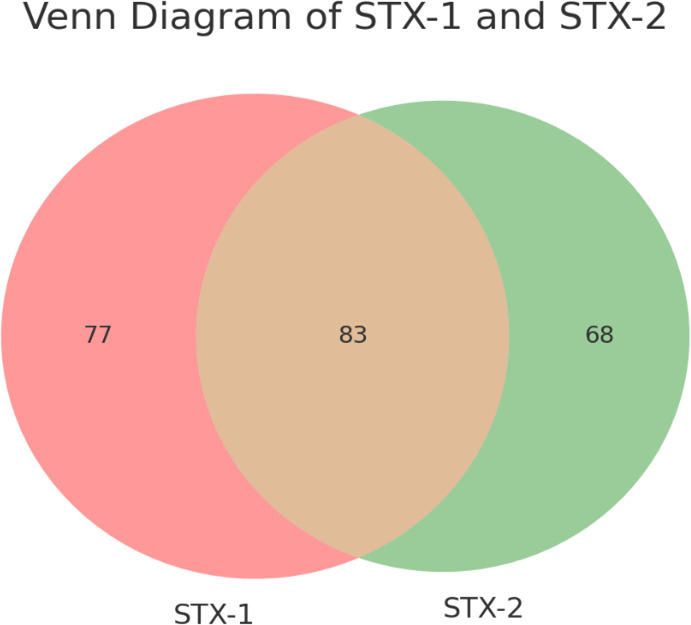
Distribution of shiga toxin gene profiles (*STX-1*, *STX-2*, and dual carriage) among 354 cattle-derived *E. coli* strains.

PCR screening of the 354 retained representative isolates revealed that 228/354 (64.4%) carried Shiga toxin genes. Specifically, 77 isolates (21.8%) were positive for stx1 alone, 68 (19.2%) were positive for stx2 alone, and 83 (23.4%) carried both stx1 and stx2 (Fig. 1).

Notably, stx2 is generally associated with more severe disease in humans (43). These prevalence values were derived from the full PCR-screened collection and are separate from the subset used for whole-genome sequencing. For WGS, we selected 11 isolates purposively rather than randomly, with the aim of capturing diversity in Shiga toxin profile, phylogroup assignment, resistance phenotype, and source type. Thus, the sequenced subset was designed for exploratory genomic characterization of a diversity-enriched panel and should not be interpreted as representative of the full 354-isolate collection.

Venn diagram showing the number of isolates positive for *stx1* only (77), *stx2* only (68), both *stx1* and *stx2* (83), and negative for both genes (126).

The prevalence of Shiga toxin genes was high, with stx1 detected in 45.2% and stx2 in 42.7% of isolates. Notably, 83 dual-positive isolates were identified within the cattle population in Dhaka. Given the human health risks associated with *STX-2*, these results underscore the importance of regular STEC monitoring and risk management in urban livestock to mitigate zoonotic transmission. Additionally, seasonal variation in stx-positive samples was observed, with prevalence ranging from 11% to 56% and higher values during late spring and summer than in winter.

To assess antibiotic susceptibility, we analyzed 65 out of 354 *E. coli* strains isolated from cattle rectal swabs and meat in Bangladesh with detailed results available in the Figshare data set (https://doi.org/10.6084/m9.figshare.32148352). All samples were collected from the Gabtoli livestock market in Dhaka. Resistance was most frequent to mecillinam and tetracyclines, with additional resistance observed to macrolides and trimethoprim–sulfamethoxazole. In contrast, resistance to ciprofloxacin, gentamicin, and colistin was uncommon, and no isolate was resistant to imipenem. These findings indicate that resistance in this collection is concentrated in a subset of antimicrobial classes, rather than being uniformly high across all drug categories.

### Genomic features and strain characterization of 11 sequenced isolates

Whole-genome sequencing was performed on 11 isolates in total. Of these, nine were obtained from rectal swabs and two were recovered from retail meat (A35 and A36). Phylogroup analysis showed that eight isolates belonged to phylogroup B1, while three (A35, A36, and B64) belonged to phylogroup A (Table 1). None of the sequenced isolates fell into phylogroup E, which is commonly associated with classical O157:H7 strains.

The inner black circle indicates the GC content and the GC skew (positive: green; negative: purple). each colored ring represents a genome from the study, with isolate identifiers labeled. Annotated regions of interest, including the stx1A and stx2B genes, are highlighted. The clustering patterns indicate genome similarity and variations among the isolates relative to the reference genome.

Phylogroup assignments determined by PCR were independently confirmed using the Clermont Typing web tool (44), yielding fully concordant results. MLST based on the *E. coli* achtman seven-locus scheme revealed substantial genetic heterogeneity both within and across phylogroups. isolates belonging to the same phylogroup frequently displayed distinct sequence types (STs) and serotypes, indicating a complex population structure.

ST155 was the most prevalent lineage and was detected in three phylogroup B1 isolates collected in April 2018 (B61, C24, and C26); notably, these strains belonged to three different serotypes (O3:H51, O153:H16, and O112:H12, respectively), demonstrating the absence of a one-to-one relationship between ST and serotype. Two phylogroup A isolates collected on 29 November 2017 (A35 AND A36) were assigned to ST6395 and shared an identical serotype (O8:H21), consistent with close clonal relatedness.

Three isolates (B59, B60, and B62) could not be assigned a known ST. These unassigned strains displayed multiple serotypes, further underscoring the genetic and antigenic diversity within this group. Among them, B62 contained a novel mdh allele closely related to allele 9 , as detailed in the Figshare data set (https://doi.org/10.6084/m9.figshare.32148352), resulting in an incomplete MLST profile, whereas B59 and B60 possessed complete allelic profiles that did not match any existing ST in the PubMLST database, suggesting previously unreported allelic combinations. Similarly, strain B64 (ST176) also exhibited multiple serotypes, reinforcing the lack of a strict correspondence between MLST-defined lineages and serotype.

**TABLE 1:** Genomic and epidemiological characteristics of the 11 *E. coli* isolates analyzed in this study^*a*^

Strain ID	Date of isolation	Strain name	Genome size (bp)	No. of contigs	*N*_50_ (bp)	GC content (%)	Phylo-group	MLST	Serotype	Source
A35	29.11.17	JZ-26A	4,694,942	197	93,123	50.74	A	ST-6395	O8:H21	Cattle (meat)
A36	29.11.17	T-J 9B-3	4,694,252	181	93,123	50.74	A	ST-6395	O8:H21	Cattle (meat)
B59	21.11.17	NGCS-240	5,813,219	891	65,510	50.31	B1	Unknown	Multiple	Cattle (rectal swab)
B60	21.11.17	NGCS-258	5,508,010	503	82,048	50.37	B1	Unknown	Multiple	Cattle (rectal swab)
B61	21.11.17	NGCS-265	4,815,410	434	68,801	50.62	B1	ST-155	O3:H51	Cattle (rectal swab)
B62	22.11.17	NGCS-267	5,493,038	597	59,308	50.46	B1	Unknown	Multiple	Cattle (rectal swab)
B64	22.11.17	NGCS-271	4,956,027	681	38,455	50.73	A	ST-176	Multiple	Cattle (rectal swab)
C24	08.04.18	NGCS-411	4,806,727	105	193,453	50.71	B1	ST-155	O153:H16	Cattle (rectal swab)
C25	08.04.18	NGCS-416A	4,658,939	90	166,342	50.71	B1	ST-3765	O153:H30	Cattle (rectal swab)
C26	08.04.18	NGCS-419	5,202,911	282	232,937	50.74	B1	ST-155	O112:H12	Cattle (rectal swab)
C27	08.04.18	NGCS-427B	5,306,791	330	113,341	50.53	B1	ST-295	O22:H16	Cattle (rectal swab)

^*a*^ A total of 352 cattle rectal swabs and 2 meat samples were collected at Gabtoli livestock market (Dhaka, Bangladesh). Eleven isolates were selected for whole-genome sequencing (nine from rectal swabs, two from retail meat). Selection aimed to represent both stx-positive and stx-negative profiles. The table shows isolate ID, source, serotype, sequence type (MLST), and phylogroup assignment (21).

### Core-genome and accessory-genome analyses place Bangladeshi cattle isolates within diverse *E. coli* lineages

**Fig 2:**
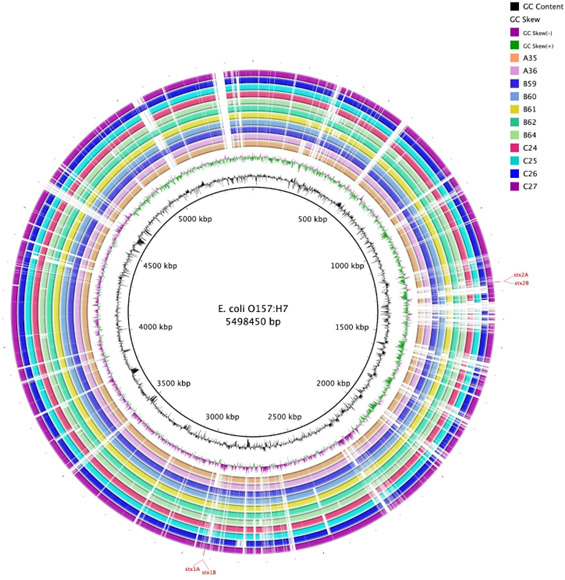
Circular genome map of E. coli O157:H7 reference at 5498450 bp, showing alignment of 11 non-O157 isolates with GC content, GC skew tracks, and stx gene markers annotated.

**Fig 3:**
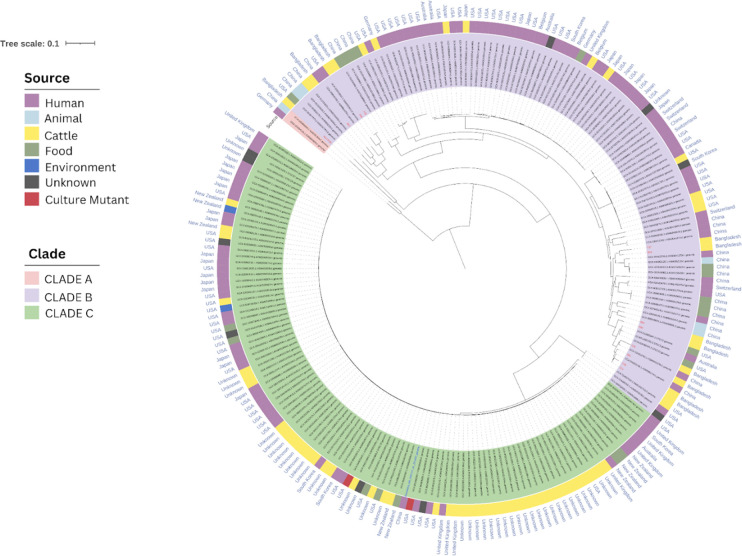
Core-genome SNP phylogeny of Bangladeshi cattle-derived *E. coli* isolates with global stx-positive *E. coli* genomes.

We performed a phylogenetic analysis on 212 publicly available *E. coli* genomes containing the *stx* gene (Fig. 2) , along with 11 genomes from our study, utilizing Snippy for core genome alignment and FastTree (31) to construct a whole-genome phylogenetic tree (Fig. 3). The list of genomes used for this analysis is available in the Figshare (https://doi.org/10.6084/m9.figshare.32148352). This analysis revealed distinct clustering patterns based on SNPs relative to the reference genome O157:H7 strain EDL933. In the resulting phylogenetic tree, genomes from NCBI are indicated in black, our study genomes in red, and the reference genome in blue. All 11 cattle isolates from this study clustered within a single major clade (Clade B), whereas the O157:H7 reference strain EDL933 fell into a separate clade (Clade C). Strains C27 and B59, which carry the stx gene and were isolated from cattle, exhibit close phylogenetic ties to two human-derived stx-positive genomes, GCA 030413785.1 and GCA 030413745.1. This pattern indicates that Bangladeshi cattle-derived isolates fall near public genomes from human or food-associated contexts in the broader *E. coli* phylogeny. However, because the public comparison set is source-heterogeneous and lacks matched temporal and epidemiological metadata, these relationships should be interpreted as shared genomic relatedness rather than evidence of direct transmission.

Additionally, B60, another stx-containing strain examined in our study, clusters with both stx-positive and stx-negative genomes found in various contexts, including food sources such as spinach (STEC2) and ground beef (STEC10). This genetic connection to strains found in food supplies across different regions underscores the adaptability of stx-positive strains in diverse environments and emphasizes the public health danger they pose in foodborne transmission pathways (45). To complement the core SNP phylogeny, we performed accessory-genome comparison using Roary. The pangenome analysis identified 30,107 total gene clusters, including 2,720 core genes, 396 soft-core genes, 3,736 shell genes, and 23,255 cloud genes. This large accessory-gene fraction indicates substantial gene-content diversity across the analyzed *E. coli*. The accessory-genome results therefore support the interpretation that the data set contains diverse lineages with variable gene content, rather than a single homogeneous cattle-associated cluster. However, no similar epidemiological linkage or outbreak investigation framework was available; we did not define a strict SNP-distance threshold for transmission. Instead, we provide the full pairwise SNP-distance matrix to allow transparent assessment of genomic relatedness.

The *stx*-negative genomes analyzed in this study (A35, A36, B61, B64, C24, and C25) did not segregate into a single cattle-specific clade. Instead, they intermingled with *E. coli* lineages associated with humans, food, and plants, suggesting shared ancestries and the potential for gene flow across hosts. Genomes C24, C25, C26, and B61 show close genetic relationships to human-sourced stx-positive genomes GCA 030413765.1 and GCA 009951745.1 from China. Despite lacking the *stx* gene, these genomes are genetically similar to stx-positive strains, suggesting either a potential for *stx* gene acquisition or a shared lineage where *stx* was lost or not gained (46). The association of bovine-derived stx-negative strains with human stx-positive strains implies that cattle may serve as reservoirs for genetic lineages capable of acquiring virulence genes under suitable conditions, raising concerns about zoonotic transmission pathways (47). Additionally, stx-negative genomes A35, A36, B61, B64, C24, and C25 did not segregate into a single cattle-specific clade but were interspersed among lineages associated with humans, food, and environmental sources (46). For instance, A35 and A36 cluster with the human-derived GCA 003018,815.1. This overlap supports the possibility of zoonotic interactions, further indicating gene flow or convergent evolution across various settings (47).

Maximum-likelihood tree inferred from a Snippy core-genome SNP alignment of 11 isolates from this study (red), 212 publicly available stx-positive *E. coli* genomes (black), retrieved from NCBI (Figshare: https://doi.org/10.6084/m9.figshare.32148352), and the O157:H7 reference strain EDL933 (blue). Bangladeshi isolates cluster within one major clade (Clade B), while EDL933 falls within a separate clade (Clade C). The tree was generated using FastTree under the GTR model, and branch support was assessed using SH-like local support values. Pairwise SNP distances were calculated from the same core SNP alignment and are provided in the Figshare data set. The tree is intended to show contextual genomic placement and shared lineage relatedness, not direct transmission.

### Virulence factor distribution and genotype–phenotype correlation

**Fig 4:**
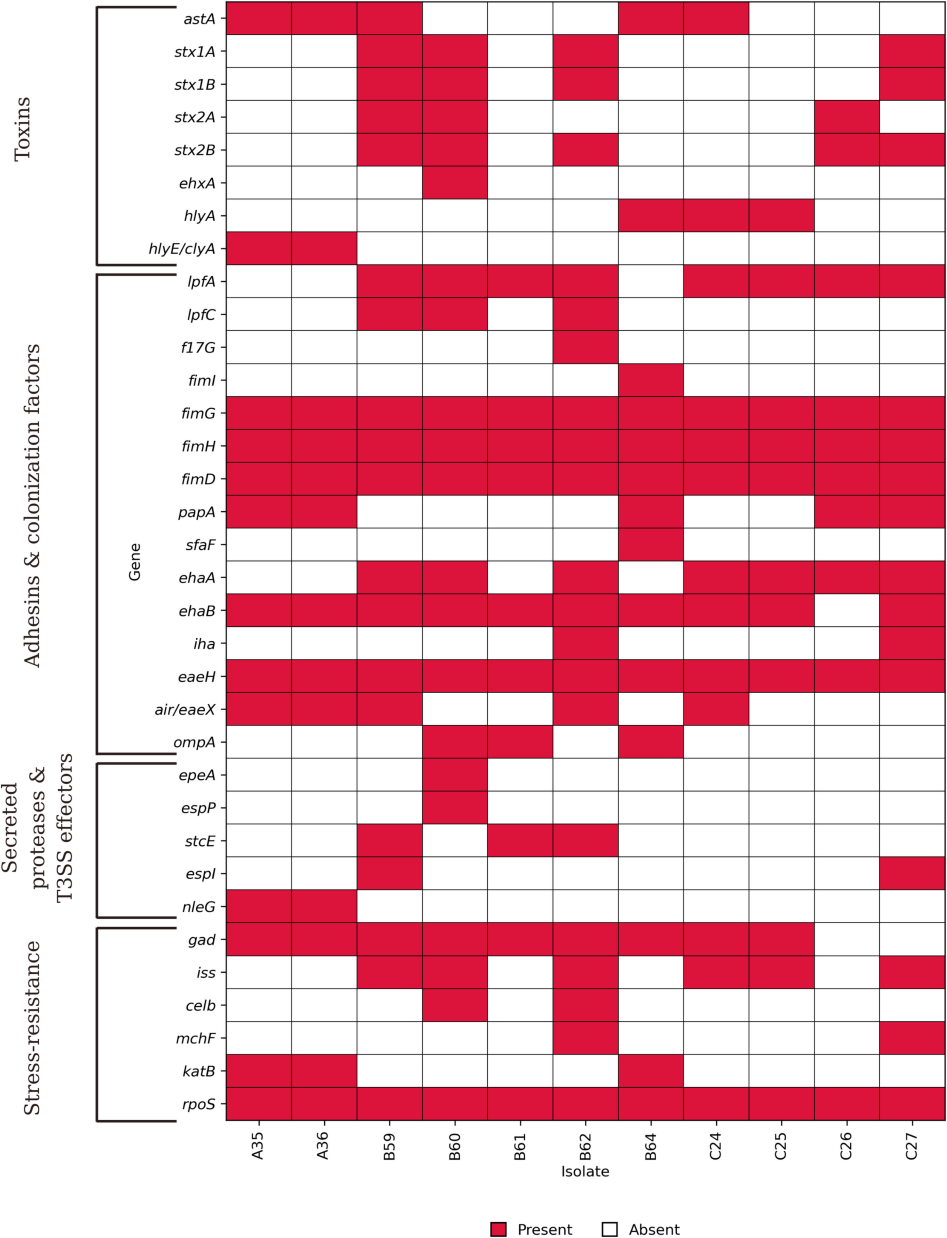
Distribution of virulence-associated genes among cattle-derived *Escherichia coli* isolates.

Across 11 *E. coli* isolates, 34 virulence-associated genes were found (Fig. 4). Phylogroup analysis identified eight isolates as B1 and three (A35, A36, and B64) as phylogroup A, which aligned with *in silico* typing (Table 1). The number of virulence genes varied between strains, with B62 having 20 genes and B61 and C26 having only 10. All isolates had five genes: eaeH (an adhesin separate from the standard intimin gene eae), fimG, fimH, fimD, and rpoS (Fig. 4). Notably, none of the genomes contained eae, a characteristic of O157 EHEC, indicating that the universally found *eaeH* should not be conflated with eae (48).

Several virulence factors were unique to phylogroup A isolates. All three phylogroup A isolates (A35, A36, and B64) carried katB (catalase–peroxidase), while hlyE/clyA and nleG were detected only in A35 and A36. Strain B64 harbored fimI and sfaF (S-fimbrial adhesin), which were not present in other isolates (Fig. 4). Five phylogroup B1 isolates (B59, B60, B62, C26, and C27) had Shiga toxin genes (stx1A, stx1B, stx2A, and/or stx2B; Fig. 4). B62 and C27 lacked stx2A; however, all five were positive for stx2B. B59 and B60 had both stx1 and stx2 variations, indicating increased virulence potential, whereas C26 carried an stx2 locus (stx2A and stx2B), which are substantially associated with severe disease outcomes (49). A comparison of total virulence gene counts with the number of acquired AMR genes showed only a weak, strain-specific correlation (Fig. 4). It indicates that the virulence and AMR burdens in this collection are not linked to each other.

Heatmap showing the presence and absence of 34 virulence genes across 11 isolates, grouped by phylogroup. Phylogroup B1 isolates predominantly carry *stx* genes, whereas phylogroup A isolates harbor distinct non-*stx* virulence factors. Core adhesin and stress-response genes are conserved across all isolates, while virulence gene content varies independently of antimicrobial resistance burden.

### Genotype–phenotype discordance in antimicrobial resistance markers

All sequenced isolates carried the multidrug efflux gene mdf(A), which confers resistance to multiple drug classes (50). Phenotypically, all strains were mecillinam resistant, yet β-lactamase genes were not detected in B59, B60, B62, B64, C25, and C26, suggesting alternative mechanisms such as porin alterations, efflux contributions, or uncharacterized β-lactam resistance pathways (51). Piperacillin resistance was observed in A35, A36, B59, B60, B61, and C24, although no bla genes were detected in B59 or B60. Notably, B59 and B60 were resistant to multiple β-lactams despite lacking detectable β-lactamase genes, again supporting noncanonical mechanisms (51). Four β-lactam-resistant strains (A35, A36, C24, and C27) carried blaTEM-1B, while B61 carried multiple other β-lactamase genes excluding blaTEM-1B.

**Fig 5:**
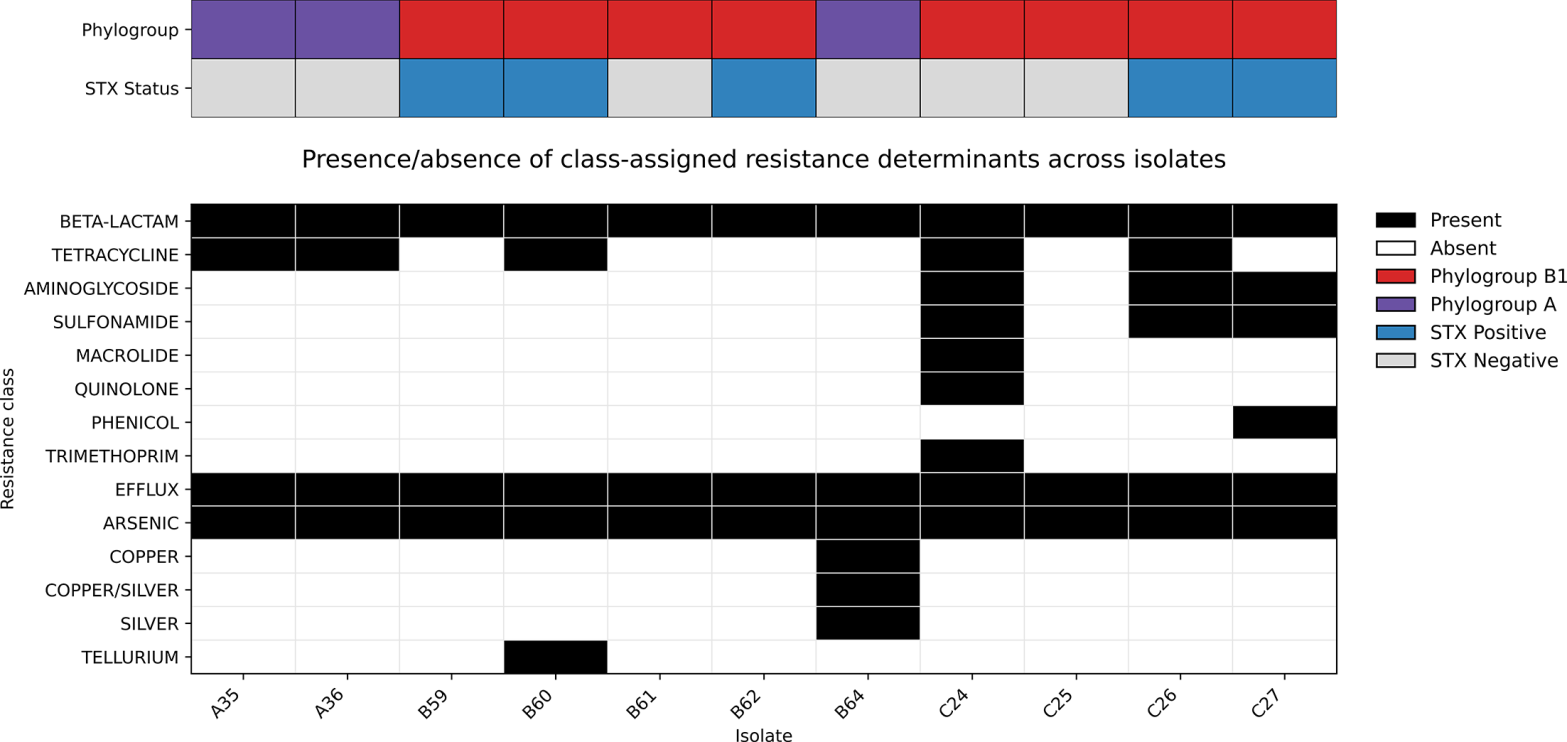
Presence/absence of class-assigned resistance determinants across sequenced *E. coli* isolates.

Heatmap shows the presence (black) or absence (white) of resistance determinants in each resistance class for 11 cattle-derived genomes. Isolates are annotated by phylogroup (A, purple; B1, red) and STX status (positive, blue; negative, gray). Additional discordance was observed for quinolone determinants: C24 carried qnrS13 but remained ciprofloxacin susceptible, whereas A35 was nalidixic acid resistant without a quinolone-specific determinant, consistent with possible efflux-linked or chromosomal mechanisms. Tetracycline resistance was common phenotypically, while several resistant isolates carried tet(A) or tet(B), B61 and B62 were doxycycline resistant despite lacking detectable tetracycline resistance genes, suggesting alternative mechanisms or regulatory effects. Other resistance determinants detected included strA/strB (streptomycin), sul2/sul3 (sulfonamides), dfrA14 (trimethoprim), and catA1 (chloramphenicol), supporting a mixed resistance repertoire in the sequenced subset. Overall, the heatmap highlights a conserved core of efflux- and arsenic-associated determinants across genomes, while antibiotic-class determinants are patchily distributed and do not segregate strictly by STX status (Fig. 5).

### Serum resistance and biofilm formation phenotypes

**Fig 6:**
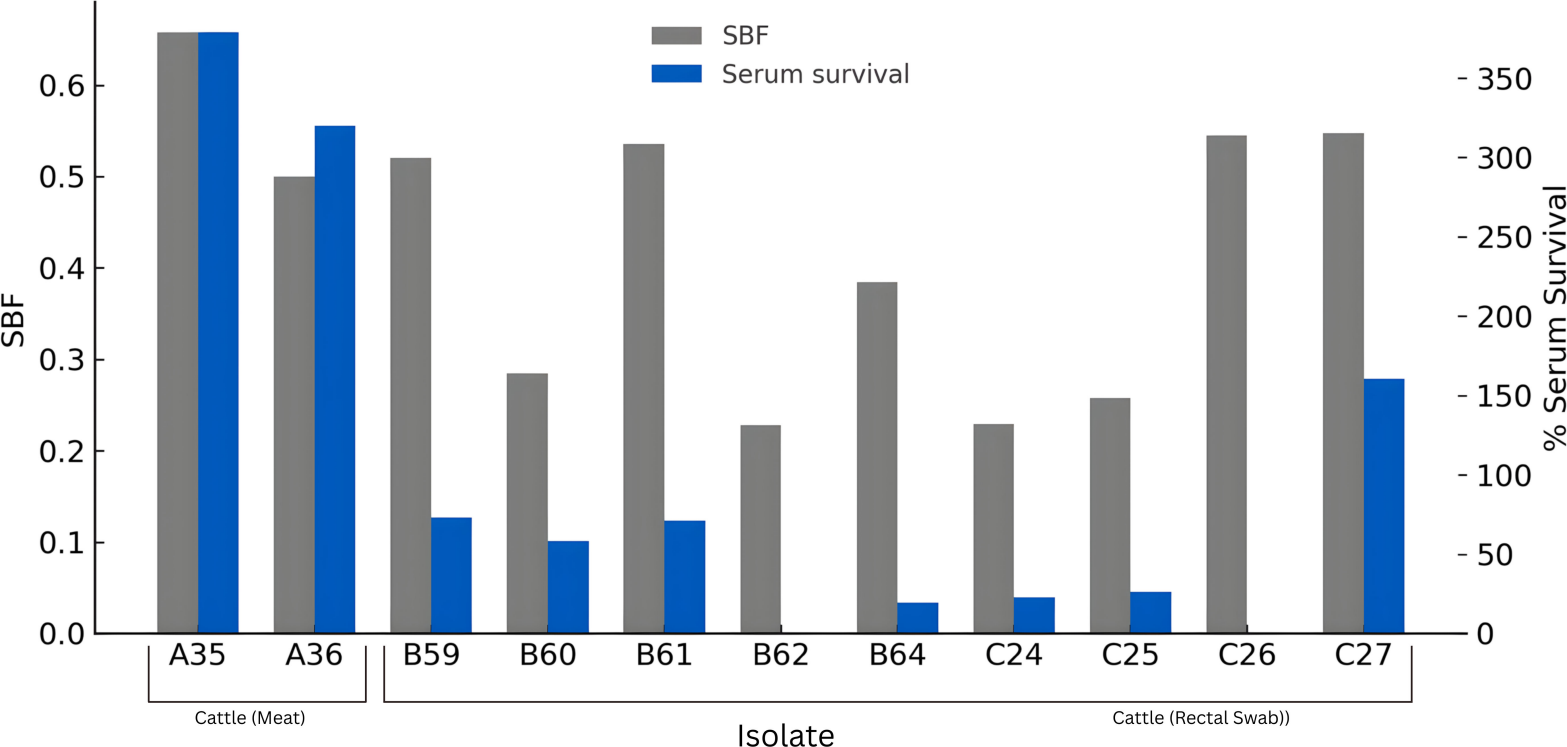
Serum resistance and biofilm-forming capacity of cattle-derived *E. coli* isolates.

Phenotypic assays for serum resistance and biofilm formation were performed only on the 11 (9 were serum sensitive) sequenced isolates and describing the exploratory WGS subset rather than the full cattle-derived isolates. The sequenced isolates exhibited phenotypic heterogeneity in serum resistance and biofilm development. While only A35 and C27 had significant survival rates (Fig. 6). To explore a potential genetic basis, we screened genomes for the serum-associated gene *iss* (increased serum survival) and related outer-membrane factors. *iss* was detected in 6/11 genomes, with inferred copy numbers ranging from 1 to 5; however, *iss* presence and copy number did not track the phenotype. Notably, B62 carried multiple *iss* copies yet remained serum sensitive, whereas A35 showed the highest serum survival despite lacking detectable *iss* (Fig. 6).

**Fig 7:**
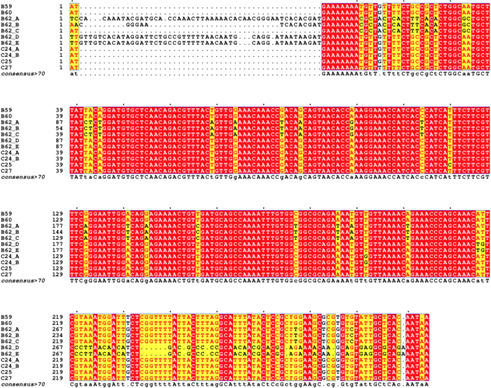
Comparative alignment of iss sequences among selected non-O157 *E. coli* isolates.

Comparative alignment of *iss* loci showed strong conservation across the coding region among *iss*-positive genomes, but clear divergence in the 5′ upstream region (Fig. 7). Variation in this upstream segment, approximately −58 to −10 relative to the start of the coding sequence, included short insertions, deletions, and sequence polymorphisms. This pattern is consistent with differences in promoter or untranslated region architecture, or with local genomic rearrangements, that could influence *iss* transcription and its contribution to complement resistance. Notably, isolate B62, the only *iss*-positive strain that was phenotypically susceptible to serum, contained a distinct insertion in this upstream region that was not present in other isolates. This provides a plausible regulatory explanation for why B62 remained serum sensitive despite carrying multiple *iss* copies.

Biofilm formation enhances bacterial persistence in the host by promoting evasion of immune clearance and survival in hostile environments. Bacteria associated with chronic infections, such as urinary tract infections, have been shown to create biofilms that aid in their infection. All of the bacterial isolates in this study formed weak to moderate biofilms; thus, no bacterial isolate was classified as forming a strong biofilm (Fig. 6). Five strains (A35, B59, B61, C26, and C27) were classified as moderate biofilm formers, while the remaining isolates were weak biofilm producers. Biofilm strength showed only a weak correlation with serum resistance (*R*² = 0.44, based on isolates with complete paired measurements, *n* = 10): both serum-resistant strains (A35 and C27) produced moderate biofilms, whereas the highly serum-sensitive strains (B62 and C26) differed in biofilm formation capacity, indicating that biofilm formation alone does not predict serum resistance (52).

Biofilm-associated genes were widely but unevenly distributed. The type 1 fimbrial cluster (*fim*), a key determinant of surface adherence (53), was present in all genomes, including the major adhesin gene *fimH* (54) and its partners *fimG* and *fimD*; only *fimI* varied, being detected exclusively in B64, a weak biofilm former. Additional biofilm-associated loci were found at a low frequency. The *hlyA* hemolysin gene was found in B64, C24, and C25, which are all weak biofilm producers. A35, A36, B64, C26, and C27 all had the *papA* gene, which encodes the principal subunit of P fimbriae. Three of these (A35, C26, and C27) developed moderate biofilms. However, all genomes lacked *papC,* which is linked to P-fimbrial-mediated biofilm formation (53). The S-fimbrial adhesin gene *sfaF* was found only in B64, which also had limited biofilm formation. The distribution of biofilm-associated genes did not consistently explain differences in biofilm phenotype or serum resistance, indicating the multifactorial nature of these features.

Serum survival and biofilm-forming propensities of 11 *E. coli* isolates. Pooled normal human serum (NHS) was inoculated with overnight LB culture at a 1:9 (vol/vol) ratio. Bacteria were enumerated at 0 and 3 h of incubation at 37°C, and percentage survival in serum was calculated. For biofilm assay, bacteria were grown in LB media, and specific biofilm formation (SBF) (second metric plotted for each strain) was calculated.

Overall, iss presence and inferred copy number did not reliably predict serum survival (Fig. 6). For example, A35 showed the highest serum survival despite lacking detectable iss, whereas B62 carried multiple copies but was serum susceptible. These findings indicate that serum resistance in this collection is multifactorial and cannot be explained by iss alone. Additional determinants, including capsule or lipopolysaccharide features, other outer membrane components, and broader complement evasion mechanisms, likely contribute to the resistant phenotypes observed in A35 and C27. Although ompA was detected in all 11 isolates, its universal presence was not associated with increased serum survival, supporting the conclusion that strain-dependent serum resistance reflects combined genetic and regulatory influences rather than iss, ompA, or virulence gene counts in isolation (Fig. 4, 6 and 7).

Comparative alignment of iss sequences is shown at the top. Across the 11 genomes screened, iss was detected in six isolates (B59, B60, B62, C24, C25, and C27), with multiple copies in B62 (five copies) and C24 (two copies). Conserved positions are highlighted by shading (predominantly red), while polymorphic sites appear as yellow; dots represent gaps. A consensus sequence (>70% threshold) is shown below each alignment block. Despite sequence conservation among iss-positive isolates/copies, iss did not consistently correlate with serum survival phenotypes (Fig. 6), supporting a multifactorial basis of complement resistance.

### Mobilome structure and horizontal transfer potential across isolates

**Fig 8:**
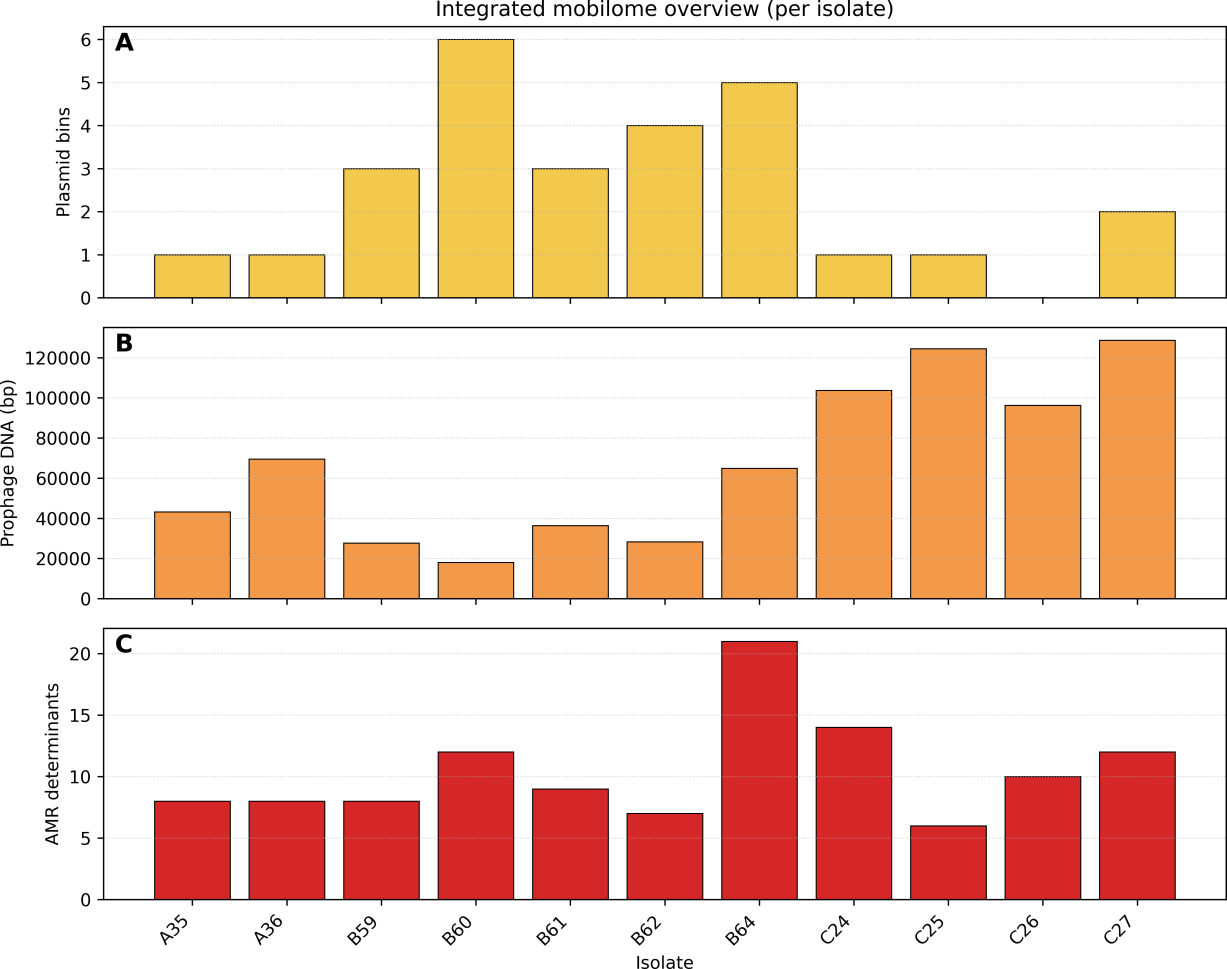
Integrated mobilome overview across 11 *E. coli* isolates. Bar charts show (A) plasmid bins, (B) prophage DNA in bp, and (C) AMR determinants per isolate across 11 E. coli strains. Isolate B64 peaks at 21 AMR determinants, while C24 and C27 lead in prophage DNA.

**Fig 9:**
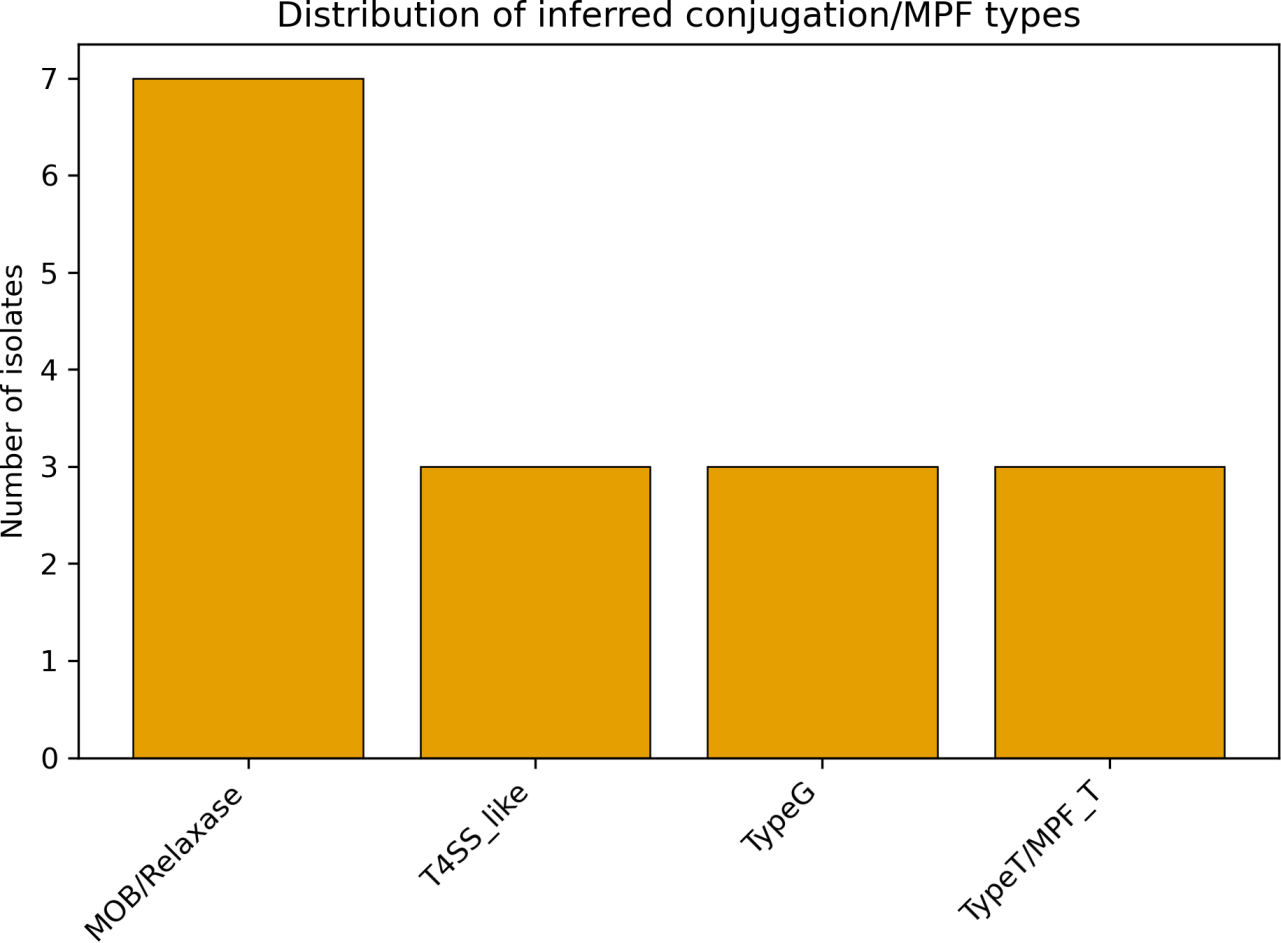
Distribution of inferred conjugation system types across isolates.

To contextualize resistance and virulence profiles (including stx carriage; Fig. 4), we quantified key mobilome features across the 11 sequenced genomes, including reconstructed plasmid bins, conjugation-associated gene clusters, prophage-like sequence content, and class-assigned nonvirulence resistance determinants (Fig. 8 and 9; Figshare: https://doi.org/10.6084/m9.figshare.32148352). Reconstructed plasmid burden varied from 0 to 6 bins per isolate (27 bins total), with the highest predicted plasmid loads in B60 (6 bins) and B64 (5 bins) and no plasmid bins detected in C26(Fig. 8A). Prophage-like sequence content estimated using PhiSpy (39) as total predicted prophage DNA on contigs ≥20 kb ranged from 18,030 to 128,629 bp (Fig. 8B) and did not segregate by stx status: high prophage burdens were observed in both stx-positive C27 and stx-negative C25/C24, indicating that the prophage pool in this collection extends beyond stx-converting elements.

Resistance determinants ranged from 6 to 21 per genome (115 total class-assigned determinants) (C). The highest determinant burden occurred in the stx-negative isolate B64 (21 determinants), whereas stx-positive genomes carried intermediate burdens (7–12), indicating that resistance gene load is not restricted to stx-positive backgrounds in this dataset (Fig. 8C) (Figshare: https://doi.org/10.6084/m9.figshare.32148352). Across isolates, the resistance-associated pool was dominated by multidrug efflux and metal/metalloid tolerance determinants, while broader antibiotic-class profiles (e.g., aminoglycoside, sulfonamide, tetracycline, quinolone, macrolide/phenicol, and trimethoprim determinants) were concentrated in a subset of genomes, including C24 and C27 (Fig. 8C).

Conjugation system screening further showed that inferred transfer machinery varied independently of reconstructed plasmid bins (Fig. 9) (Figshare: https://doi.org/10.6084/m9.figshare.32148352). MOB/relaxase signatures were most frequently detected (7/11 genomes), whereas T4SS-like and MPF class signals (Type G and Type T/MPF_T) were restricted to smaller subsets of isolates (Fig. 9). Notably, C26 and C27 harbored the highest number of CONJScan systems (10 each) despite carrying zero and two reconstructed plasmid bins, respectively, consistent with conjugation-related modules that may be chromosomal or integrated and therefore not recovered as discrete plasmid bins in fragmented assemblies. IntegronFinder (38) did not detect integrons or attC sites in any of the 11 genomes, suggesting that integron-mediated cassette arrays were not a major feature of the mobilome in this isolate set.

Bars indicate the number of isolates in which each conjugation-associated feature was detected. Inferred systems include relaxase-associated mobility signatures (MOB/relaxase), type IV secretion system–like components (T4SS-like), and mating-pair formation (MPF) system classes (Type G and Type T/MPF_T). Detection was based on genome-wide screening for conjugation-related gene clusters.

Across the studied isolates, an apparent inverse association between reconstructed plasmid bins and predicted prophage DNA burden was observed (Spearman *ρ* = −0.66, *P* = 0.028), alongside a positive association with non-antibiotic stress/metal resistance determinants (*ρ* = 0.68, *P* = 0.022) (Fig. 8). However, several genomes in this study were represented by fragmented draft assemblies, with some isolates containing more than 500 contigs (Table 1), which may limit the accurate recovery and assignment of mobile genetic elements. In such cases, plasmid sequences and prophage regions may be split across contigs or incompletely assembled, potentially leading to misclassification or partial detection. Therefore, the observed associations may partly reflect assembly-dependent artifacts rather than true biological relationships and should be interpreted cautiously.

## DISCUSSION

Genetic data on Shiga toxin–producing *E. coli* (STEC) from Bangladesh remain scarce, particularly for non-O157 strains in livestock (9). In this study, we combined PCR-based screening of 354 cattle-derived isolates with whole-genome sequencing and phenotypic characterization of 11 representative non-O157 *E. coli*, including both stx-positive and stx-negative strains. Our analysis provides baseline insights into stx distribution among cattle in a major urban livestock market, defines the genomic and virulence characteristics of non-O157 strains, and underscores the presence of significant antimicrobial resistance. This study’s findings highlight the critical need to combine genetic and phenotypic surveillance to understand the epidemiology and public health impact of STEC in Bangladesh (9, 55).

Across the 65 isolates examined, resistance was universal to mecillinam and was frequently observed for tetracyclines and macrolides. In contrast, resistance to ciprofloxacin, gentamicin, and colistin was uncommon, and no isolate showed resistance to carbapenems. Similar resistance patterns have been reported in other low- and middle-income settings, where older and widely used antibiotics often show reduced effectiveness, while activity of last-line agents remains largely preserved (56–58). This trend likely reflects extensive antimicrobial use in livestock production and highlights the need to strengthen antimicrobial stewardship and routine resistance surveillance in food animal systems in Bangladesh. Because this subgroup was selected descriptively rather than randomly, these findings should be interpreted as patterns observed in the tested isolates rather than as prevalence estimates of the entire collection.

Our mobilome analysis provides a genomic context for these resistance patterns and suggests that resistance and persistence traits in these genomes are shaped by multiple mobile element classes rather than by a single dominant platform. Across the 11 sequenced isolates, reconstructed plasmid burden ranged from 0 to 6 plasmid bins per isolate, while total predicted prophage DNA varied more than sevenfold, and class-assigned nonvirulence resistance determinants ranged from 6 to 21 per isolate (Fig. 8). Notably, plasmid burden and resistance determinant burden were not tightly coupled, and plasmid-rich isolates did not uniformly exhibit high resistance gene counts. This observation aligns with broader trends reported globally in non-O157 *E. coli*, where accessory gene content is often structured by lineage, local selection pressures, and ecological exposures rather than plasmid carriage alone. In addition, the predominance of efflux- and metal/metalloid-tolerance determinants in the genomic resistance pool suggests that non-antibiotic selection pressures (including metals and disinfectants used in agriculture and food environments) may contribute to maintenance of accessory gene cargo alongside antimicrobial use.

We detected a range of virulence genes among the sequenced isolates, including *stx1* and *stx2* in a subset of strains and multiple adhesins such as *fimH* and *fimG*, indicating the potential to cause disease(59). However, the existence of virulence genes does not imply expression or clinical pathogenicity *in vivo*. Many non-O157 STEC strains lack the classical intimin gene (*eae*) and are regarded to have less pathogenic potential (9). Therefore, none of our isolates carried eae; instead, all of them encoded the similar adhesin *eaeH*, type 1 fimbrial genes, and the stress-response regulator *rpoS.* Because *eaeH* is not functionally similar to eae and may not confer the same pathogenic potential as regular EHEC, these genomic profiles should be taken as indications of virulence potential rather than conclusive evidence of high pathogenicity in humans (48). Comparative studies also show that specific stx subtypes, particularly *stx2a*, are strongly associated with hemolytic uremic syndrome (HUS) (60), whereas other subtypes may carry lower risk (61); thus, detailed stx subtyping is critical for risk assessment. Furthermore, stx subtype analysis was not performed, which limits fine-scale interpretation of virulence potential, particularly as some subtypes, such as stx2a, are more strongly associated with severe human disease than others (60, 61). As a result, our genomic findings should be interpreted as indicators of virulence-associated gene content rather than precise predictors of clinical severity.

Whole-genome phylogenetic analysis linked the Bangladeshi cattle isolates to globally distributed *E. coli* lineages that contain both human and food-derived stx-positive strains (3). The majority of our sequenced isolates belonged to phylogroups B1 and A, which are typically associated with environmental and human pathogenic *E. coli,* which are commonly associated with environmental and human-related *E. coli* lineages (62). Notably, the stx-positive isolates C27 and B59 clustered with human-derived stx-positive genomes, whereas B60 clustered with a mix of stx-positive and stx-negative strains from dietary sources like spinach and ground beef. Similarly, numerous stx-negative isolates from our study (such as A35, A36, C24, and C25; particularly A35 and A36, which differed by only 22 SNPs) mixed with human and plant-associated lineages rather than creating a cattle-specific clade. These findings support genomic contextualization of the Bangladeshi isolates but do not demonstrate direct transmission. The public genome set was heterogeneous with respect to source, geography, and collection metadata, and the analysis was not designed as an outbreak or transmission investigation. Therefore, we interpret these relationships as evidence of shared lineage structure and broad genomic relatedness across cattle, human, food, and environmental contexts (45–47).

Accessory-genome analysis also indicated extensive pangenome diversity. Roary identified 30,107 total gene clusters, of which only 2,720 were core genes, while 23,255 belonged to the cloud genome. This large accessory component is consistent with the open and diverse population structure expected for *E. coli* and supports the observation that gene content varies substantially across lineages. Therefore, both core SNP and accessory-genome analyses support the conclusion that the analyzed isolates represent genetically diverse *E. coli* backgrounds rather than a uniform cattle-associated clone.

We found descriptive seasonal variation in stx-positive isolates over the nine sampling rounds, with higher proportions during late spring and summer and lower proportions during winter. Although this pattern should be taken with caution due to the data set’s short time depth and number of sample rounds, it is broadly comparable with findings from Europe and North America indicating higher STEC shedding or infection rates during warmer months (59, 63–66). Multiple factors, such as higher ambient temperatures, changes in diet and management, and increased livestock movement and trade during various seasons, can all contribute to transmission and environmental persistence. Recognizing seasonality allows for targeted treatments such as improved hygiene and biosecurity measures, concentrated surveillance, and risk communication during periods of elevated transmission (67).

Importantly, we found multiple incidences of genotype-phenotype discordance. Some isolates carried acquired AMR genes but remained phenotypically susceptible, whereas others demonstrated resistance in the absence of detectable resistance determinants. B59 and B60 were resistant to β-lactams while lacking β-lactamase genes, whereas C24 had qnrS13 but was susceptible to ciprofloxacin. Such inconsistencies could be due to context-dependent gene expression, compensatory mutations, chromosomal resistance mechanisms, or limitations in discovering novel or divergent resistance genes in current databases (51, 64, 68). These observations highlight that WGS-based prediction of resistance must be interpreted alongside phenotypic testing.

A similar pattern appeared when we examined serum resistance. The presence or copy number of *iss*, and the occurrence of ompA, did not consistently predict survival in normal human serum. For example, B62 had several *iss* copies but was still vulnerable to serum, whereas A35 had no *iss* but demonstrated great survival. Only A35 and C27 were clearly serum resistant, while having differing *iss* and biofilm characteristics. These findings are consistent with past research demonstrating that complement resistance in *E. coli* relies on the combined action of many surface features, including capsules, outer membrane proteins such as OmpA, and the LPS O antigen, as well as regulatory pathways, rather than a single factor like *Iss* (69, 70).

Beyond plasmid bins and prophage burden, our MGE screening suggests that horizontal transfer capacity may also be influenced by conjugation machinery encoded outside reconstructed plasmid bins. Conjugation-associated system detection revealed marked isolate-level variation, including isolates with no detectable systems and others with strong signals spanning relaxase-associated signatures and MPF system types (Fig. 9). Notably, C26 and C27 carried the highest number of inferred conjugation-associated systems, consistent with the presence of chromosomally encoded or integrated transfer modules that may not assemble into discrete plasmid bins in fragmented draft genomes. In contrast, integron screening was uniformly negative across all 11 genomes (no integrons and no attC sites), suggesting that integron-mediated cassette capture is unlikely to be a major driver of accessory gene acquisition in this isolate set under the present assemblies and detection thresholds. Together, these findings support a model in which plasmids and prophage-like regions represent major variable components of the mobilome, while conjugation modules may exist independently and integrons appear uncommon in this collection. Because several genomes remained highly fragmented draft assemblies, structural inferences regarding plasmid bins, prophage burden, and their interrelationships should be interpreted cautiously. In particular, plasmid reconstruction from fragmented assemblies may misclassify chromosomal regions or incompletely recover true plasmid structure.

Our study has several important limitations. First, all samples were collected from a single large urban livestock market (Gabtoli Hat in Dhaka), which may not fully represent cattle populations from rural farms or other regions of Bangladesh.

Second, only 11 isolates were sequenced, which limits the strength of genomic inference and statistical power. In addition, prevalence estimates were derived from PCR screening of one retained representative isolate per sample; because within-sample diversity was not exhaustively characterized, the reported 64.4% value should be interpreted cautiously as an isolate-based estimate rather than a direct measure of sample-level heterogeneity. Third, prevalence estimates were based on PCR screening of one representative *E. coli* isolate per sample; because within-sample colony diversity was not exhaustively characterized, some mixed-population samples (containing both stx-positive and stx-negative colonies) could be under- or over-represented. Fourth, the observed seasonal trends are based on nine sampling rounds and are given descriptively; longer-term monitoring with rigorous statistical testing will be required to evaluate whether these patterns persist over time. Given these constraints, our findings should be seen as hypothesis-generating baseline data that will need to be confirmed in larger, multisite research.

Future research should build on these findings by expanding monitoring to various livestock markets and agricultural settings throughout Bangladesh, increasing the number and diversity of non-O157 *E. coli* and STEC isolates subjected to WGS. Larger data sets will allow for more robust statistical analyses of the relationships between genetic characteristics, AMR profiles, and clinical or environmental data. In parallel, host-pathogen interaction studies, including transcriptome analysis during infection, will be critical for understanding how unique virulence gene combinations and regulatory circuits influence colonization, serum resistance, and toxin production *in vivo* (71). Integrating such data into a One Health framework, complementing human clinical and environmental surveillance, would be critical in developing effective, region-specific treatments to lower the incidence of STEC and other pathogenic *E. coli* in Bangladesh (43). Together, these findings suggest that high-throughput monitoring at large urban livestock markets such as Gabtoli Hat could serve as an early warning point for *E. coli* lineages and mobile gene pools that may enter downstream food and human exposure pathways.

### Conclusion

This study provides a detailed genomic and phenotypic characterization of non-O157 *E. coli*, including STEC, circulating in Bangladeshi cattle. It provides essential baseline data on the prevalence and seasonality of stx carriage, genomic diversity, and virulence gene content in cattle-associated lineages, and the widespread presence of multidrug resistance determinants.

However, the small sequenced subset, reliance on a single sampling site, and descriptive analyses limit the generalizability of our findings.

The core SNP and accessory-genome analyses placed the Bangladeshi cattle-derived isolates within diverse *E. coli* genomic backgrounds and showed substantial variation in accessory gene content across the comparison set. These results support the relevance of cattle-derived *E. coli* within a broader One Health genomic context, while also emphasizing that direct transmission cannot be inferred without matched epidemiological and source-linked data.

Future studies should expand multi-site WGS surveillance, and integrating genomic data with phenotypic assays such as AMR testing, biofilm formation, and serum resistance will be essential to refine risk assessments and guide control strategies. Strengthening One Health surveillance and antibiotic stewardship in the cattle sector will contribute to reducing the public health threat posed by emerging zoonotic pathogens (72).

## Data Availability

The sequencing data supporting this study have been deposited in the GenBank under the following accession numbers: JBJCIP000000000, JBJCIO000000000, JBJCIN000000000, JBJCIM000000000, JBJCIL000000000, JBIYZJ000000000, JBIYZI000000000, JBIYZH000000000, JBIYZG000000000, JBIYZF000000000, and JBIYZD000000000. Raw sequencing reads for the 11 sequenced isolates have been deposited in the NCBI Sequence Read Archive (SRA) under BioProject accession PRJNA1182077. All datasets generated during this study are publicly available in Figshare. Additional phylogenomic and accessory-genome outputs generated using Snippy and Roary have also been deposited in Figshare (https://doi.org/10.6084/m9.figshare.32148352).
